# How Is Colorectal Cancer Care Impacted by Global Crisis in Contrasting Healthcare Systems?—A Descriptive Study From Scotland and Switzerland During the COVID‐19 Pandemic

**DOI:** 10.1002/wjs.70294

**Published:** 2026-03-02

**Authors:** B. Wiesler, M. Worni, P. Studer, J.‐M. Gass, J. Metzger, M. Hartel, C. Nebiker, R. Rosenberg, R. Galli, L. Eisner, C. Andreou, U. Zingg, D. Stimpfle, C. T. Viehl, A. Müller, B. Müller, K. Denhaerynck, P. Hall, C. Gallagher, P. Karunaratne, C. Lilley, M. Zuber, H. Paterson, M. von Strauss und Torney

**Affiliations:** ^1^ Clarunis, Department of Visceral Surgery University Digestive Health Care Center, St. Clara Hospital and University Hospital Basel Basel Switzerland; ^2^ Stiftung Lindenhof I Campus SLB, Swiss Institute for Translational and Entrepreneurial Medicine Berne Switzerland; ^3^ Department of Visceral Surgery Cantonal Hospital of Lucerne Lucerne Switzerland; ^4^ Department of Health Sciences and Medicine University of Lucerne Lucerne Switzerland; ^5^ Department of Visceral Surgery Cantonal Hospital of Aarau Aarau Switzerland; ^6^ Department of Visceral Surgery Cantonal Hospital of Basel‐Land Liestal Switzerland; ^7^ Department of Surgery Cantonal Hospital of Olten Olten Switzerland; ^8^ Department of Visceral Surgery Spital Limmattal Schlieren Switzerland; ^9^ Department of Surgery Spitalzentrum Biel Biel Switzerland; ^10^ Department of Public Health University of Basel Basel Switzerland; ^11^ Edinburgh Cancer Research Centre University of Edinburgh Edinburgh Scotland; ^12^ St. Clara Research Ltd., St. Clara Hospital Basel Switzerland; ^13^ Department of Colorectal Surgery Western General Hospital Edinburgh Scotland

**Keywords:** centralization, colorectal cancer, pandemic

## Abstract

**Background:**

Healthcare across Europe was affected by COVID‐19 pandemic lockdowns. How different national healthcare systems coped with this impact remains unclear. Healthcare in Switzerland differs significantly from that in Scotland, for example, in terms of centralization. The aim of this study was to assess the impact of the COVID‐19 pandemic on the diagnosis and surgical treatment of colorectal cancer (CRC) in contrasting healthcare systems.

**Patients and Methods:**

This retrospective cohort study was conducted in south–east Scotland and in the extended north–west of Switzerland from January 1st, 2019 to February 28th, 2023. All patients diagnosed with CRC were included. The primary outcomes were the time from CRC diagnosis to treatment and the UICC stage at diagnosis, assessed prior to, during, and following the period of lockdown. The lockdown in Scotland lasted from March 2020 to October 2020 and in Switzerland from March 2020 to April 2020.

**Results:**

A total of 6745 patients were included (4127 from Scotland and 2618 from Switzerland). Median time from diagnosis to treatment remained unaltered during the lockdown period in both countries. However, after the lockdown, the median time from diagnosis to treatment increased from 59 to 76 days in Scotland. The median number of patients who were diagnosed per annual quarter declined from 177 (IQR: 171–190) to 152 (IQR: 150–154), and the median number of who received treatment from declined from 256 (IQR: 253–259) to 203 (IQR: 186–218) during lockdown in Scotland. In multivariable logistic regression, the odds of being diagnosed with UICC stage IV increased by 42% for patients diagnosed during lockdown (95%‐CI: 12%–81%). In Switzerland, the time from diagnosis to treatment increased slightly after the pandemic. However, the other effects described above were not observed in Switzerland.

**Conclusions:**

This descriptive study demonstrated that the impact of the pandemic on colorectal cancer care was less pronounced in Switzerland, but considerable in Scotland. Because separate subgroup analyses were conducted, direct comparisons cannot be made between Scotland and Switzerland.

**Trial registration:**

This trial is registered on clinicaltrials.gov as part of the EvaCol study (NCT04550156)

## 1 Background

The global impact of the COVID‐19 pandemic on surgical oncology was tremendous. Approximately 2.3 million cancer operations were postponed during the initial phase [1]. Ninety‐six percent of colorectal surgeons reported that the COVID‐19 pandemic had affected their clinical practice [2]. Restrictions were imposed on both surgical treatment and screening of colorectal cancer (CRC) patients. In the United Kingdom, the COVID‐19 pandemic led to the interruption of most non‐COVID‐19 services, resulting in a 92% reduction of the performed colonoscopies from April 2020 to October 2020 [3, 4, 5]. Available evidence suggests that elective surgery for CRC should not be delayed by more than 4 weeks, as longer delays are associated with poorer outcomes [6]. It is therefore reasonable to assume that during the COVID‐19 pandemic patients were at increased risk of disease progression due to delay in diagnosis and treatment.

In comparison to many European healthcare systems, the Scottish healthcare system is strongly centralized, meaning almost all non‐COVID‐19 elective services were discontinued during the COVID‐19 pandemic [5]. The lockdown in Scotland started in March 2020 and lasted until October 2020 [7]. Compared to Scotland, Switzerland has a higher capacity healthcare system consisting of numerous small units, which allowed many non‐COVID‐19 services to continue despite the pandemic, with minimal disruptions at a smaller number of sites and availability of continued services at neighboring hospitals. The discrepancies between the two healthcare systems are exemplified by the divergence in the number of hospital beds per capita and the related expenditure. The number of beds per 1000 is 2.4 in Scotland compared with 4.4 in Switzerland [8]. Health expenditure accounts for 11.1% of gross domestic product (GDP) in Scotland (population 5.5 million), equivalent to US$19.98 billion, and 11.3% of GDP in Switzerland (population 9 million), equivalent to US$105.61 billion [9, 10, 11, 12]. It is also crucial to consider the delivery of care in small outpatient practices, which have historically been the primary entities responsible for ongoing screening activities in Switzerland. Due to the higher density of hospitals and the high‐capacity CRC care in the Swiss health care system, the hospitals in Switzerland were able to ensure the care of patients even in the event of a higher incidence of COVID‐19 cases, without ending up in a triage situation. This was one of the reasons why the lockdown in Switzerland, which started in March 2020, lasted only 6 weeks [13].

We hypothesized that the extended lockdown and cessation of screening colonoscopies in Scotland would be associated with more advanced CRC stage at diagnosis, and that this would be prevented or at least less impactful in the high‐capacity healthcare system of Switzerland. The aim of this study was to describe changes in colorectal cancer care during the COVID‐19 pandemic in both systems. Therefore, the delay in diagnosis and treatment of patients in the two health care systems and the impact of this delay on tumor stage at diagnosis were assessed.

## 2 Patients and Methods

This retrospective cohort study was conducted in nine hospitals in the extended north–west of Switzerland (NWS), and in the south–east of Scotland (SES), both covering a population of similar size. The catchment area in Switzerland is difficult to define precisely, due to the overlap with other hospitals that were not included in the study. However, it can be estimated to be between 1.0 and 1.2 million inhabitants. All patients diagnosed with CRC at one of the participating hospitals between January 1st, 2019 and February 28th, 2023 were included. Patients with recurrence of CRC were excluded. In Scotland, the south–east Scotland Cancer Network (SCAN) quality performance indicators (QPI) colorectal cancer dataset was utilized, which includes all patients diagnosed with CRC from four Scottish health boards (Lothian, Fife, Borders, and Dumfries & Galloway; population 1.46 million). This prospectively collected dataset was linked with an extract of National Records of Scotland (NRS) death data to derive 30‐day mortality, and to the Scottish inpatient and day‐case records (SMR01) to derive Charlson Comorbidity Index using the ICD‐10 diagnoses recorded within 5 years prior to the diagnosis (Supporting Information S1: Figure S1). The linkage was performed using the Community Health Index (CHI) unique patient identifier. In north–west Switzerland, the nine participating hospitals cover all CRC patients treated in the region, with the exception of outpatients treated in private gastroenterology practices (although these patients are usually referred to hospitals at some point for treatment planning) and those who opt out of further use of their data. Another exception is the Lindenhofspital in the canton of Berne, where some patients may have had treatment in other facilities in the canton. Patients were identified retrospectively from the German Cancer Society Cancer Registry, which includes all patients with a confirmed diagnosis of CRC. For centers not certified by the German Cancer Society, patients were selected by ICD codes C18, C19, and C20 from the hospital information systems. Baseline data was obtained from the report submitted to the Federal Statistical Office (BFS), which is a mandatory requirement for all hospitals in Switzerland. Missing data was obtained from medical records and the dataset of the EvaCol study [14]. Follow‐up for at least 30 days was performed. The study was conducted following the Strengthening the Reporting of Observational Studies in Epidemiology (STROBE) reporting guideline [15]. It was approved by the local ethics committee “EKNZ” (Ethikkommission Nordwest‐und Zentralschweiz) on September 28th, 2021, under ethic number 2020‐01494.

## 3 Outcomes and Data Collection

The primary objectives of the study were to assess the extent of diagnostic and therapeutic delay during lockdown in the two countries and its impact on tumor stage at diagnosis. The secondary objectives were the number of patients who were diagnosed and treated within the healthcare region before, during, and after lockdown, the 30‐day postoperative mortality rate, and the stoma rate.

The dataset included patient and procedural characteristics (age, sex, Charlson Comorbidity Index (CCI), treatment type, presentation type, and surgical approach), oncological data (date of diagnosis, TNM classification, use of preoperative or postoperative chemoradiation, date of surgical, or nonsurgical treatment), and postoperative outcome data (date of death). Variables were extracted from the various data sources and transferred to a database hosted on a secure server at NHS Scotland in Edinburgh, Scotland.

The national lockdown due to COVID‐19 in Switzerland was declared on 16th of March 2020 and lasted until 27th of April 2020 (6 weeks) [13]. In Scotland, the national lockdown started in 23rd of March 2020 and ended on 12th of October 2020 (30 weeks) [7]. We defined the *pre‐lockdown* period from the 1^st^ January, 2019 to the start of the respective country's lockdown period. We defined the *lockdown* period as per each country's duration of lockdown plus 2 months to account for re‐establishment of services, and the *post‐lockdown* period from then to 28th February, 2023.

## 4 Statistical Analysis

Descriptive analysis used appropriate summary statistics (median, interquartile range (IQR), frequencies, and percentages) to describe patient and procedural characteristics and outcomes for different lockdown phases in each country separately.

Logistic regression analysis was conducted to investigate the relationship between lockdown periods with two outcomes: 30‐day mortality, and diagnosis of advanced colorectal cancer (UICC stage‐IV). Univariable logistic regression included age, sex, CCI, localization of cancer, presentation, neoadjuvant therapy, time to first treatment, and lockdown period. Multivariable logistic regression for 30‐day mortality incorporated all statistically significant variables in univariable analysis, with the exception of time to first treatment. Multivariable analysis for the diagnosis of advanced CRC excluded neoadjuvant therapy and time to first treatment. Multiple imputation was used as over 10% of the data were missing. This was done using the “mice” package in R. The level of statistical significance was set at a *p*‐value of ≤ 0.05 and all *p*‐values were calculated using a two‐sided test. All analyses were conducted using R statistical software, version 4.1.3 (2002). Because separate subgroup analyses were conducted, direct comparisons cannot be made between Scotland and Switzerland.

## 5 Results

A total of 4127 patients were included from the south–east of Scotland (SES group) and 2618 from the extended north–west of Switzerland (NWS group). Median age did not differ between the groups. There was a higher proportion of men in both groups (Table 1). The ratio of patients presenting as emergencies increased during the lockdown period in both groups. Although the proportion of patients who underwent laparoscopic surgery increased from the pre‐lockdown period to the lockdown period in the SES group, there was a trend toward open surgery in the NWS group during lockdown (Table 1). A detailed comparison of patient and oncological characteristics is shown in Supporting Information S1: Table S1.

**TABLE 1:** Patient, oncological, and procedural characteristics, overall and subdivided by country.

	SES group (*n* = 4127)	NWS group (*n* = 2618)	Total (*n* = 6745)
Age (median, IQR)	72 (63–80)	71 (61–79)	72.0 (62–80)
Men (*n*, %)	2198 (53.3%)	1509 (57.7%)	3707 (55.0%)
Charlson comorbidity index (*n*, %)			
0	1356 (54.8%)	1289 (54.8%)	2645 (54.8%)
1–2	612 (24.7%)	325 (13.8%)	937 (19.4%)
≥ 3	506 (20.4%)	738 (31.4%)	1244 (25.8%)
Localization (*n*, %)			
Colon	3046 (73.8%)	1654 (66.9%)	4700 (71.2%)
Rectum	1081 (26.2%)	819 (33.1%)	1900 (28.8%)
Neoadjuvant therapy (*n*, %)	331 (8%)	404 (15.4%)	735 (10.9%)
Adjuvant therapy (*n*, %)	767 (18.6%)	335 (12.8%)	1102 (16.3%)
Palliative therapy (*n*, %)	580 (14%)	292 (11.2%)	872 (12.9%)
Type of the first therapy (*n*, %)			
Surgery	2500 (60.6%)	1700 (64.9%)	4200 (62.2%)
Radiotherapy	242 (5.9%)	80 (3.1%)	322 (4.8%)
Chemotherapy	78 (1.9%)	225 (8.6%)	303 (4.5%)
Radiochemotherapy	97 (2.3%)	275 (10.5%)	372 (5.5%)
None	1210 (29.3%)	338 (12.9%)	1548 (23.0%)
Any type of therapy received (*n*, %)			
Surgery	3125 (75.7%)	2105 (80.4%)	5230 (77.5%)
Radiotherapy	291 (7%)	389 (14.9%)	680 (10.1%)
Chemotherapy	1072 (26%)	548 (20.9%)	1620 (24.0%)
Radiochemotherapy	116 (2.8%)	293 (11.2%)	409 (6.1%)
None	762 (18.5%)	325 (12.4%)	1087 (16.1%)
Emergency presentations (*n*, %)			
Pre lockdown	166 (18.5%)	198 (28.9%)	364 (23.0%)
During lockdown	108 (26.7%)	51 (32.5%)	159 (28.3%)
Post lockdown	281 (17.4%)	310 (26.5%)	591 (21.2%)
Open procedures (*n*, %)			
Pre lockdown	439 (35.2%)	364 (45%)	803 (39.1%)
During lockdown	166 (28.5%)	107 (54.3%)	273 (35.0%)
Post lockdown	631 (27.5%)	769 (47.7%)	1400 (35.8%)
Laparoscopic procedures (*n*, %)			
Pre lockdown	421 (33.8%)	238 (29.5%)	659 (32.1%)
During lockdown	219 (37.6%)	51 (25.9%)	270 (34.7%)
Post lockdown	726 (31.6%)	544 (33.7%)	1270 (32.5%)
Robotic procedures (*n*, %)			
Pre lockdown	0 (0%)	9 (1.1%)	9 (0.5%)
During lockdown	0 (0%)	4 (2%)	4 (0.5%)
Post lockdown	169 (7.4%)	52 (3.2%)	221 (5.6%)

Abbreviations: NWS, North‐West Switzerland; SES, South‐East Scotland.

### 5.1 Primary and Secondary Outcomes

**FIGURE 1:**
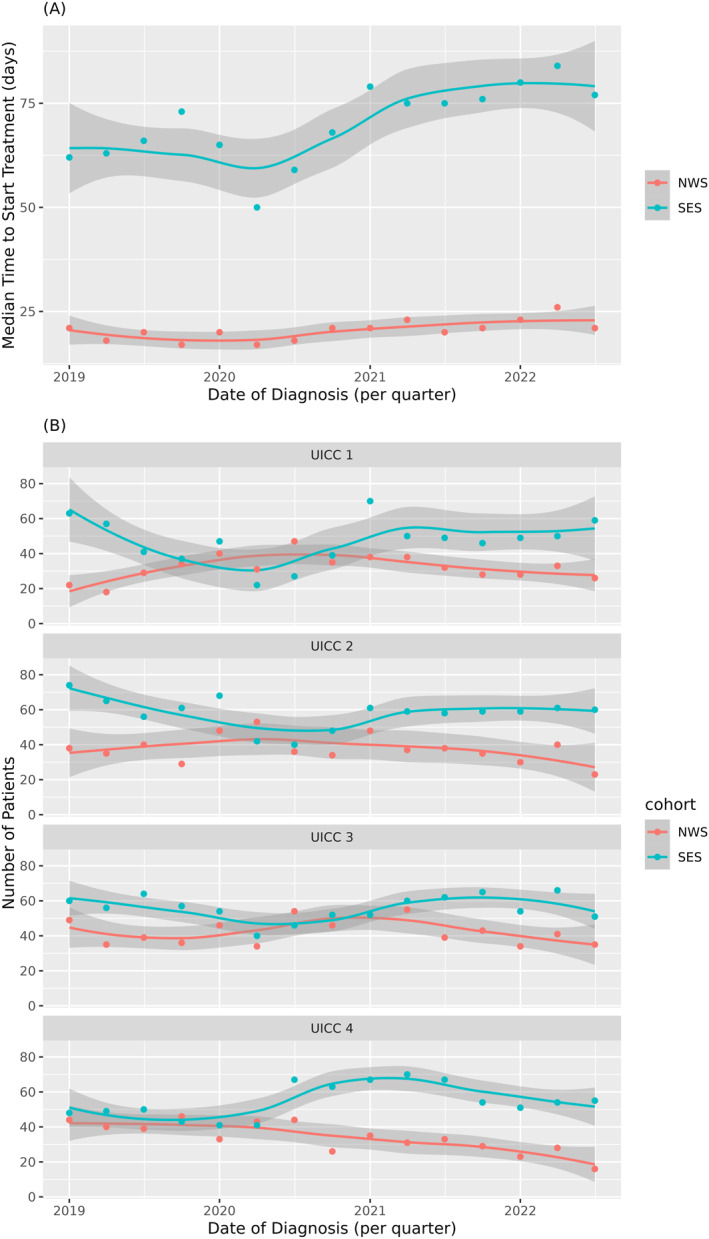
Results of the primary endpoints: (A) Time from diagnosis to first treatment. (B) Number of patients diagnosed with different UICC stages per quarter. NWS, north–west Switzerland; SES, south–east Scotland.

Figure 1A shows the median time from diagnosis to treatment per quarter for each group. The median time to start treatment did not differ in the SES and NWS groups when comparing the pre‐lockdown and lockdown periods. However, after the lockdown period ended, the median time to start treatment increased in the SES group (Supporting Information S1: Table S2). Median time to start treatment per quarter subdivided by elective and emergency patients is shown in the online resources Supporting Information S1: Figure S2. The number of patients diagnosed with UICC stage I and II cancer decreased during lockdown in the SES group. Conversely, the number of patients diagnosed with UICC stage IV cancer increased during lockdown (Figure 1B and Supporting Information S1: Table S2). The NWS group did not exhibit an increase in the number of patients diagnosed with advanced stages during lockdown (Figure 1B and Supporting Information S1: Table S2). The distribution of T‐, N‐, and M‐stages in the SES and NWS groups is illustrated in the online resources Supporting Information S1: Figure S3.

**FIGURE 2:**
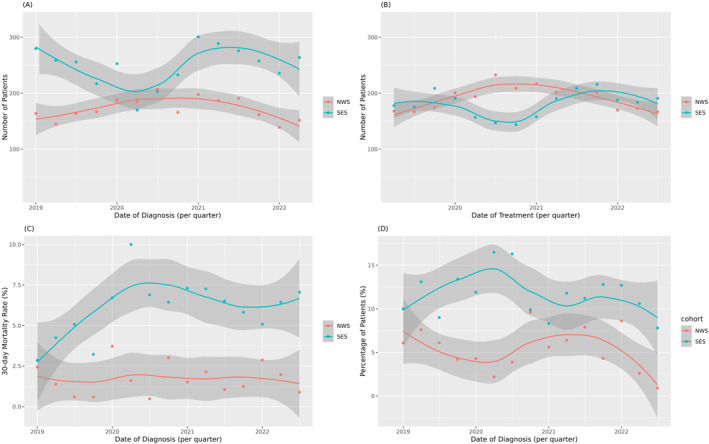
Results of the secondary endpoints: (A) Number of diagnosed patients per quarter. (B) Number of treated patients per quarter. (C) 30‐day mortality rate. (D) Permanent Stoma rate. NWS, north–west Switzerland; SES, south–east Scotland.

The number of newly diagnosed and treated patients per quarter in the SES group decreased during the lockdown period. This was followed by an increase in these categories after the lockdown ended. In contrast, the NWS group did not experience a decrease in the number of patients diagnosed or treated during the lockdown period (Figure 2A–B, Supporting Information S1: Table S3). The 30‐day mortality rate increased from 4% to 8% in the SES group during the lockdown period and remained at 2% in the NWS group (Figure 2C, Supporting Information S1: Table S3). Permanent stoma rate increased during the lockdown period in the SES group (Figure 2D, Supporting Information S1: Table S3).

### 5.2 Univariable and Multivariable Logistic Regression

In univariable logistic regression, age, higher CCI, emergency cases, and diagnosis during lockdown were associated with a higher probability of being diagnosed with advanced cancer in the SES group (Table 2). In the multivariable logistic regression model, the odds for being diagnosed with advanced cancer was found to be 42% higher for individuals in SES who were diagnosed during the lockdown period (95% CI: 12%–81%) (Table 2). In the NWS group, however, being diagnosed during the lockdown period was not associated with an elevated risk of being diagnosed with advanced cancer. Table 3 shows the results for 30‐day mortality, following multiple imputation. In multivariable logistic regression analysis, being diagnosed during lockdown was not associated with an increased likelihood of death within 30 days in either country (Table 3). Supporting Information S1: Tables S4 and S5 present additional complete case analyses for the two outcomes.

**TABLE 2:** Univariable and multivariable logistic regression analysis for being diagnosed with advanced tumor stage (UICC IV) in Scotland (SES group) and Switzerland (NWS group) following multiple imputation for missing data.

SES group		*n*	Unadjusted odds ratio	95% CI	*p* value	Adjusted odds ratio	95% CI	*p* value
Age		4127	1.01	1.00–1.02	**0.002**	1.00	0.99–1.01	0.86
Sex	Female	1929	Reference					
	Male	2198	1.01	0.88–1.17	0.85	—	—	—
CCI	0	2406	Reference			Reference		
	1–2	953	1.19	0.99–1.42	0.06	1.04	0.86–1.26	0.69
	≥ 3	768	3.74	3.15–4.45	**<** **0.001**	2.44	2.01–2.95	**<** **0.001**
Localization	Colon	3046	Reference			Reference		
	Rectum	1081	0.77	0.65–0.90	**0.002**	1.35	1.12–1.63	**0.001**
Presentation	Elective	3075	Reference			Reference		
	Emergency	1052	5.88	5.05–6.87	**<** **0.001**	5.24	4.43–6.21	**<** **0.001**
Pandemic	Pre‐lockdown	1247	Reference			Reference		
	Lockdown	582	1.71	1.38–2.13	**<** **0.001**	1.42	1.12–1.81	**0.004**
	Post‐lockdown	2298	1.23	1.04–1.44	**0.014**	1.30	1.09–1.55	**0.004**
Time to first treatment		4127	0.99	0.99–0.99	**<** **0.001**	—	—	—

NWS group		*n*	Unadjusted odds ratio	95% CI	*p* value	Adjusted odds ratio	95% CI	*p* value
Age		2618	0.99	0.99–1.00	**0.016**	0.99	0.98–0.99	**<** **0.001**
Sex	Female	1106	Reference					
	Male	1512	0.92	0.77–1.11	0.38	—		
CCI	0	1456	Reference			Reference		
	1–2	349	0.84	0.59–1.19	0.35	0.85	0.59–1.21	0.39
	≥ 3	813	5.03	4.11–6.17	**<** **0.001**	4.94	4.02–6.10	**<** **0.001**
Localization	Colon	1753	Reference			Reference		
	Rectum	865	0.79	0.65–0.96	**0.021**	0.89	0.72–1.10	0.29
Presentation	Elective	1911	Reference			Reference		
	Emergency	707	2.38	1.96–2.88	**<** **0.001**	2.28	1.85–2.82	**<** **0.001**
Pandemic	Pre‐lockdown	808	Reference			Reference		
	Lockdown	197	0.89	0.62–1.27	0.53	1.03	0.69–1.50	0.90
	Post‐lockdown	1613	0.72	0.60–0.88	**0.001**	0.78	0.63–0.97	**0.024**
Time to first treatment		2618	1.00	1.00–1.01	**<** **0.001**	—	—	—

*Note:* Bold values indicate *p* values above significance thresholds.

Abbreviations: CCI, charlson comorbidity index; CI, confidence interval; NWS, North‐West Switzerland; SES, South‐East Scotland.

**TABLE 3:** Univariable and multivariable logistic regression analysis for 30‐day mortality after operation in Scotland (SES group) and Switzerland (NWS group) following multiple imputation for missing data.

SES group (number of death = 248)		*n*	Unadjusted odds ratio	95% CI	*p* value	Adjusted odds ratio	95% CI	*p* value
Age		4127	1.05	1.04–1.07	**<** **0.001**	1.03	1.02–1.05	**<** **0.001**
Sex	Female	1929	Reference					
	Male	2198	0.86	0.66–1.11	0.23	—	—	—
CCI	0	2406	Reference			Reference		
	1–2	953	1.84	1.31–2.58	**<** **0.001**	1.31	0.91–1.87	0.14
	≥ 3	768	4.08	3.03–5.52	**<** **0.001**	1.93	1.39–2.68	**<** **0.001**
Localization	Colon	3046	Reference			Reference		
	Rectum	1081	0.40	0.27–0.57	**<** **0.001**	1.15	0.74–1.74	0.52
Presentation	Elective	3075	Reference			Reference		
	Emergency	1052	12.04	8.92–16.50	**<** **0.001**	9.15	6.61–12.86	**<** **0.001**
Neoadjuvant	No	3796	Reference			Reference		
	Yes	331	0.13	0.03–0.35	**0.001**	0.44	0.11–1.26	0.18
Pandemic	Pre‐lockdown	1247	Reference			Reference		
	Lockdown	582	1.90	1.26–2.84	**0.002**	1.35	0.87–2.08	0.17
	Post‐lockdown	2298	1.52	1.11–2.11	**0.010**	1.60	1.15–2.26	**0.007**
Time to first treatment		4127	0.98	0.98–0.99	**<** **0.001**	—	—	—

NWS group (number of death = 43)		*n*	Unadjusted odds ratio	95% CI	*p* value	Adjusted odds ratio	95% CI	*p* value
Age		2618	1.06	1.03–1.09	**<** **0.001**	1.04	1.01–1.07	**0.005**
Sex	Female	1106	Reference					
	Male	1512	1.53	0.82–2.99	0.20	—	—	—
CCI	0	1456	Reference			Reference		
	1–2	349	1.94	0.68–4.95	0.18	1.47	0.51–3.80	0.445
	≥ 3	813	3.38	1.74–6.86	**<** **0.001**	2.87	1.46–5.88	**0.003**
Localization	Colon	1753	Reference			Reference		
	Rectum	865	0.26	0.09–0.61	**0.005**	0.55	0.19–1.29	0.21
Presentation	Elective	1911	Reference			Reference		
	Emergency	707	2.89	1.57–5.32	**0.001**	1.96	1.05–3.66	**0.034**
Neoadjuvant	No	2214	Reference			Reference		
	Yes	404	0.00	0–2.5 × 10^10^	0.99	0.00	0–9.3 × 10^9^	0.99
Pandemic	Pre‐lockdown	808	Reference			Reference		
	Lockdown	197	0.54	0.08–1.94	0.42	0.62	0.10–2.27	0.54
	Post‐lockdown	1613	0.87	0.46–1.68	0.66	0.98	0.52–1.93	0.96
Time to first treatment		2618	1.00	1.00–1.01	0.05	—	—	—

*Note:* Bold values indicate p values above significance thresholds.

Abbreviations: CCI, charlson comorbidity index; CI, confidence interval; NWS, North‐West Switzerland; SES, South‐East Scotland.

## 6 Discussion

This descriptive analysis of datasets representative of the Scottish (and by extension, UK) and the Swiss healthcare systems evaluating the impact of the COVID‐19 pandemic on the diagnosis and treatment of CRC patients has yielded several noteworthy findings. In Scotland, the proportion of patients diagnosed with early‐stage cancer decreased during lockdown, whereas the odds of being diagnosed with UICC‐Stage IV cancer increased by 42% during the period of lockdown. The median time from diagnosis to treatment remained unaltered during the lockdown in Scotland; however, it increased after the end of the lockdown. Moreover, a decline in the number of diagnosed and treated patients during the period of lockdown in Scotland was observed. In Switzerland, the time from diagnosis to treatment increased slightly after the pandemic. However, the other effects described above were not observed in Switzerland.

A delay in the treatment of CRC patients in various countries has been reported [16]. In contrast, there was no delay in treatment during lockdown in the south–east of Scotland, possibly a consequence of the cessation of noncancer elective services. However, the time from diagnosis to treatment increased after the lockdown, a consequence of a backlog of cases and reduced capacity persisting for many months (workforce, supply chains, etc.), which resulted in an overload of the healthcare system after the lockdown ended. The proportion of patients diagnosed with early stage CRC in Scotland declined during lockdown, consistent with the suspension of national bowel cancer screening [17]. Douglas et al. demonstrated that a 10‐month delay in diagnosis increased the probability of being diagnosed at an advanced stage of the disease [18]. The period of reduced screening colonoscopy capacity in the UK lasted 9 months [5]. In accordance with this, the proportion of patients diagnosed with advanced stages increased during the lockdown period in Scotland. A large registry‐based study of 17,938 patients confirms that there was an upstaging of CRC patients during the COVID‐19 pandemic [19]. One potential explanation for this phenomenon is that by treating fewer early‐stage cases, it became possible for more biologically aggressive cancers to progress. The observation that patients diagnosed in Scotland during the pandemic exhibited a higher morbidity rate lends support to the hypothesis that these patients presented with advanced symptoms as emergency cases. This is probably attributable to the suspension of routine screening examinations in Scotland during the pandemic. Other reasons for this increase in emergency presentations include patients with symptoms finding it difficult to access primary care assessment and the fact that diagnostic services took much longer to return than the lockdown periods, not just for CRC but for all conditions. Consequently, the relative increase in emergency presentations was much higher in the SES group. The observation that the higher morbidity does not result in a higher mortality rate may be ascribed to the fact that, in contrast to disease‐specific mortality, the overall mortality can be influenced by a variety of factors. The occurrence of a higher morbidity rate does not necessarily result in a higher complication rate, nor does it necessarily result in a higher 30‐day mortality rate. It is highly probable that the consequences of upstaging will only become evident in the 3‐ or 5‐year mortality rate.

Centralization of surgical services is associated with reduced post‐operative complication rates and improved pathological outcomes, particularly in highly specialized gastrointestinal surgery [20, 21, 22, 23, 24]. Centralization is also seen as a cost‐saving measure, in part due to lower complication rates for major surgery [25]. In response to cost pressures, a significant number of healthcare systems in Europe have implemented a process of centralization [26]. Critics argue that the observed positive effect is attributable to case volume and experience of the treating surgeon, suggesting that the importance of the high‐volume center is relative [27]. From a patient’s perspective, there has been criticism of the increasing need to travel [28, 29]. A survey conducted in New Zealand revealed that the majority expressed a preference for surgery at a nearby hospital [30]. The findings of our study indicate that a high‐capacity healthcare system is more resilient in sustaining colorectal cancer care in the context of a public health emergency. Although the COVID‐19 pandemic has reached its conclusion, a new significant challenge for healthcare systems will emerge, for example, a large military conflict, economic instability, or another viral pandemic. Although in conducting this study we were particularly interested in the contrast in centralization of services, the differences between the two health systems go beyond centralization. The number of hospital beds per person differs significantly between Scotland and Switzerland. In addition, per capita healthcare expediture in Switzerland is much higher than in Scotland. The health system in Scotland is close to full capacity under normal circumstances, whereas in Switzerland there is some degree of oversupply. This assumption is supported by the fact that, even before the closure period, the time from diagnosis to treatment for elective cases was three times longer in Scotland. Indeed, one of the main reasons that lockdown in the UK was longer than in other European countries was to protect the more limited hospital capacity.

This study is subject to a number of limitations. There were missing data for some variables, which given the partial use of administrative data, could have been subject to minor miscoding. However, given the robust overall trends in treatment delays and upstaging, we believe that this did not result in major selection bias. It was not possible to conduct a multivariable analysis of treatment delays due to missing variables, such as bed and intensive care capacity, in the participating centers. The current dataset is deficient in information pertaining to oncological long‐term outcomes, including 5‐year survival and recurrence rates. Nevertheless, UICC cancer stages offer a reliable surrogate for these outcomes. Furthermore, issues were identified with the coding of data pertaining to anastomotic leakage and reoperation rates. It therefore was not possible to report these data. The lack of an official cancer registry within Switzerland necessitated the derivation of the relevant data from a variety of sources, including the extraction of some data from medical records. The utilization of three disparate data sources may have resulted in the misidentification of certain variables. In certain instances, the conflicting nature of these sources necessitated a decision regarding the prioritization of one source over another. Furthermore, the increasing number of patients treated in the NWS group across all periods may suggest a bias in the data collection. Although, the data from the federal statistical office serves as the foundation for the official Swiss hospital statistics, the potential for bias arising from data collection cannot be excluded. Moreover, the contrasting duration of lockdowns in Scotland and Switzerland and the fact that regulatory measures differed significantly between the two countries could potentially introduce another source of bias. The fact that the Swiss cohort is smaller than the Scottish cohort, although they cover a comparable health region population, may indicate that the Swiss cohort is not representative of the entire population of that region. In addition, the fact that significantly more emergency cases were observed in the Swiss group suggests that a number of patients may have been treated in other institutions. On the other hand, the much shorter time taken to initiate treatment for colorectal cancer in emergency patients in the SES group compared to the NWS group demonstrates the high level of prioritization and good organization of colorectal cancer care in Scotland. The lack of a national cancer registry in Switzerland may have resulted in patients not being included in the study because they were treated in outpatient or private clinics outside of the north–west of Switzerland, as the vast majority of patients in Switzerland have insurance cover that allows free choice of the treating institution.

This is a descriptive study describing changes in colorectal cancer care in two different healthcare systems during the COVID‐19 pandemic. One of the major differences between the Swiss and Scottish healthcare systems is the degree of centralization. However, there are other major differences between Switzerland and Scotland and their health care systems, such as population density, hospital capacity per capita, and healthcare expenditures in each country, resulting in a higher capacity within the Swiss healthcare system. In addition, the restrictions during lockdown and the length of lockdown varied significantly between countries. It was not possible to control for all these differences and it remains unclear to what extent these factors influenced our results. Further research could explore what proportion of the effects described above are due to centralization.

It should be noted, however, that the study encompasses two entire healthcare regions in Scotland and Switzerland. It can be assumed that the results are highly generalizable for the population‐based character of the study. Although this generalizability is limited to some extent by the limitations noted above, this study adds another dimension to the discussion on centralization and health care capacity. The findings of this study demonstrate that it is feasible to maintain a high standard of colorectal cancer care even in challenging circumstances such as a pandemic, provided that the healthcare system comprises a multitude of autonomous or semiautonomous units.

## 7 Conclusion

This descriptive study demonstrated that the impact of the pandemic on colorectal cancer care was less pronounced in Switzerland, but considerable in Scotland. Because separate subgroup analyses were conducted, direct comparisons cannot be made between Scotland and Switzerland. Future studies should focus on other potential contributing factors explaining such differences beyond the mode of care to balance the pros and cons of centralization, as centralization has been proven to be beneficial in many other aspects of surgical care.

## Author Contributions

**B. Wiesler:** conceptualization, data curation, funding acquisition, investigation, methodology, project administration, validation, visualization, writing – original draft. **M. Worni:** data curation, supervision, writing – review and editing. **P. Studer:** data curation, supervision, writing – review and editing. **J.‐M. Gass:** data curation, supervision, writing – review and editing. **J. Metzger:** data curation, supervision, writing – review and editing. **M. Hartel:** data curation, supervision, writing – review and editing. **C. Nebiker:** data curation, supervision, writing – review and editing. **R. Rosenberg:** data curation, supervision, writing – review and editing. **R. Galli:** data curation, supervision, writing – review and editing. **L. Eisner:** data curation, supervision, writing – review and editing. **C. Andreou:** data curation, supervision, writing – review and editing. **U. Zingg:** data curation, supervision, writing – review and editing. **D. Stimpfle:** data curation, supervision, writing – review and editing. **C. T. Viehl:** data curation, supervision, writing – review and editing. **A. Müller:** data curation, supervision, writing – review and editing. **B. Müller:** supervision, validation, writing – review and editing. **K. Denhaerynck:** investigation, resources, software, validation, writing – review and editing. **P. Hall:** conceptualization, formal analysis, investigation, methodology, validation, writing – review and editing. **C. Gallagher:** formal analysis, methodology, software, validation, visualization, writing – review and editing. **P. Karunaratne:** formal analysis, methodology, software, validation, visualization, writing – review and editing. **C. Lilley:** methodology, supervision, validation, writing – review and editing. **M. Zuber:** conceptualization, data curation, funding acquisition, investigation, methodology, supervision, validation, writing – review and editing. **H. Paterson:** conceptualization, data curation, formal analysis, investigation, methodology, project administration, supervision, validation, writing – original draft, writing – review and editing. **M. von Strauss und Torney:** conceptualization, data curation, formal analysis, funding acquisition, investigation, methodology, project administration, supervision, validation, writing – original draft, writing – review and editing.

## Funding

The study was funded by the private foundation “Krebsliga beider Basel” (KLBB) in Switzerland as part of a research grant (ID: KLbB‐5330‐03–2021). The KLBB exerted no influence on the design of the study, the collection of the data, the analysis of the data, the preparation of the manuscript and the publishing decision.

## Ethics Statement

This project has been approved by the EKNZ (Ethikkomission Nord‐West Schweiz) on September 28, 2021 under ethic number 2020‐01494.

## Consent

According to HFG Art.34, no written consent was required.

## Conflicts of Interest

The authors declare no conflicts of interest.

## Supporting information


Supporting Information S1

## Data Availability

The datasets generated and/or analyzed for this study are not publicly available, but are available from the corresponding author on reasonable request.

## References

[1] Elective Surgery Cancellations Due To the COVID‐19 Pandemic: Global Predictive Modelling to Inform Surgical Recovery Plans,” British Journal of Surgery 107, no. 11 (2020): 1440–1449, 10.1002/bjs.11746. 32395848 PMC7272903

[2] J. W. Nunoo‐Mensah , M. Rizk , P. F. Caushaj , et al., “COVID‐19 and the Global Impact on Colorectal Practice and Surgery,” Clinical Colorectal Cancer 19, no. 3 (2020): 178, 10.1016/j.clcc.2020.05.011. 32653470 PMC7276135

[3] R. D. Neal , P. Tharmanathan , B. France , et al., “Is Increased Time to Diagnosis and Treatment in Symptomatic Cancer Associated With Poorer Outcomes? Systematic Review,” supplement, British Journal of Cancer 112, no. S1 (2015): S92–S107, 10.1038/bjc.2015.48. 25734382 PMC4385982

[4] G. Del Vecchio Blanco , E. Calabrese , L. Biancone , G. Monteleone , and O. A. Paoluzi , “The Impact of COVID‐19 Pandemic in the Colorectal Cancer Prevention,” International Journal of Colorectal Disease 35, no. 10 (2020): 1951–1954, 10.1007/s00384-020-03635-6. 32500432 PMC7271141

[5] E. J. A. Morris , R. Goldacre , E. Spata , et al., “Impact of the COVID‐19 Pandemic on the Detection and Management of Colorectal Cancer in England: A Population‐Based Study,” Lancet Gastroenterology & Hepatology (2021). 10.1016/S2468-1253(21)00005-4 PMC7808901 33453763

[6] T. M. Whittaker , M. E. G. Abdelrazek , A. J. Fitzpatrick , et al., “Delay to Elective Colorectal Cancer Surgery and Implications for Survival: A Systematic Review and Meta‐Analysis,” Colorectal Disease 23, no. 7 (2021): 1699–1711, 10.1111/codi.15625. 33714235 PMC8251304

[7] Prime Minister’s Statement on Coronavirus (COVID‐19, (2020) GOV.UK, https://www.gov.uk/government/speeches/pm‐address‐to‐the‐nation‐on‐coronavirus‐23‐march‐2020.

[8] Group WB , Hospital Beds (Per 1,000 People), (2024), https://data.worldbank.org/indicator/SH.MED.BEDS.ZS?locations=GB‐CH.

[9] P. H. Scotland , Scottish Health Service Costs 2024, https://publichealthscotland.scot/publications/scottish‐health‐service‐costs/scottish‐health‐service‐costs‐summary‐for‐financial‐year‐2022‐to‐2023/#:~:text=In%20real%20terms%20total%20expenditure,billion%20in%202021%20to%202022.&text=In%20the%20financial%20year%202022%2F23%3A,billion%20spent%20in%202021%2F22.

[10] Office SFS , Costs, Financing, (2024), https://www.bfs.admin.ch/bfs/en/home/statistics/health/costs‐financing.html.

[11] Census Ss , Scotland's Census 2022—Rounded Population Estimates, (2021), https://www.scotlandscensus.gov.uk/2022‐results/scotland‐s‐census‐2022‐rounded‐population‐estimates/.

[12] Office SFS , Population at the End of the 2nd Quarter 2024, (2024), https://www.bfs.admin.ch/bfs/en/home/news/whats‐new.gnpdetail.2024‐0538.html.

[13] B. A. G. BfG , Covid‐19‐Verordnung 2 Art. 10a, (2020).

[14] B. Wiesler , R. Rosenberg , R. Galli , et al., “Effect of a Colorectal Bundle in an Entire Health Care Region in Switzerland: Results From a Prospective Cohort Study (EvaCol Study),” International Journal of Surgery (2024). 10.1097/JS9.0000000000002123 PMC11634084 39453984

[15] E. von Elm , D. G. Altman , M. Egger , S. J. Pocock , P. C. Gøtzsche , and J. P. Vandenbroucke , “The Strengthening the Reporting of Observational Studies in Epidemiology (STROBE) Statement: Guidelines for Reporting Observational Studies,” Journal of Clinical Epidemiology 61, no. 4 (2008): 344–349, 10.1016/j.jclinepi.2007.11.008. 18313558

[16] A. Mazidimoradi , F. Hadavandsiri , Z. Momenimovahed , and H. Salehiniya , “Impact of the COVID‐19 Pandemic on Colorectal Cancer Diagnosis and Treatment: A Systematic Review,” Journal of Gastrointestinal Cancer 54, no. 1 (2023): 171–187, 10.1007/s12029-021-00752-5. 34843058 PMC8628028

[17] G. Longcroft‐Wheaton , N. Tolfree , A. Gangi , R. Beable , and P. Bhandari , “Data From a Large Western Centre Exploring the Impact of COVID‐19 Pandemic on Endoscopy Services and Cancer Diagnosis,” Frontline Gastroenterology 12, no. 3 (2021): 193–199, 10.1136/flgastro-2020-101543. 33907616 PMC8040512

[18] D. A. Corley , C. D. Jensen , V. P. Quinn , et al., “Association Between Time to Colonoscopy After a Positive Fecal Test Result and Risk of Colorectal Cancer and Cancer Stage at Diagnosis,” JAMA 317, no. 16 (2017): 1631–1641, 10.1001/jama.2017.3634. 28444278 PMC6343838

[19] M. Rottoli , A. Gori , G. Pellino , et al., “Colorectal Cancer Stage at Diagnosis Before Vs During the COVID‐19 Pandemic in Italy,” JAMA Network Open 5, no. 11 (2022): e2243119, 10.1001/jamanetworkopen.2022.43119. 36409496 PMC9679872

[20] M. Rottoli , A. Spinelli , G. Pellino , et al., “Effect of Centre Volume on Pathological Outcomes and Postoperative Complications After Surgery for Colorectal Cancer: Results of a Multicentre National Study,” British Journal of Surgery 111, no. 1 (2024): znad373, 10.1093/bjs/znad373. 37963162 PMC10771132

[21] D. Leonard , F. Penninckx , A. Kartheuser , et al., “Effect of Hospital Volume on Quality of Care and Outcome After Rectal Cancer Surgery,” British Journal of Surgery 101, no. 11 (2014): 1475–1482, 10.1002/bjs.9624. 25142810

[22] E. Nugent and P. Neary , “Rectal Cancer Surgery: Volume‐Outcome Analysis,” International Journal of Colorectal Disease 25, no. 12 (2010): 1389–1396, 10.1007/s00384-010-1019-1. 20661600

[23] C. T. Aquina , C. P. Probst , A. Z. Becerra , et al., “High Volume Improves Outcomes: The Argument for Centralization of Rectal Cancer Surgery,” Surgery 159, no. 3 (2016): 736–748, 10.1016/j.surg.2015.09.021. 26576696

[24] J. D. W. Choi , T. Shepherd , A. Cao , T. El‐Khoury , N. Pathma‐Nathan , and J. W. T. Toh , “Is Centralization for Rectal Cancer Surgery Necessary?,” Colorectal Disease (2024). 10.1111/codi.17119 39107879

[25] R. Vonlanthen , K. Slankamenac , S. Breitenstein , et al., “The Impact of Complications on Costs of Major Surgical Procedures: A Cost Analysis of 1200 Patients,” Annals of Surgery 254, no. 6 (2011): 907–913, 10.1097/sla.0b013e31821d4a43. 21562405

[26] R. Vonlanthen , P. Lodge , J. S. Barkun , et al., “Toward a Consensus on Centralization in Surgery,” Annals of Surgery 268, no. 5 (2018): 712–724, 10.1097/sla.0000000000002965. 30169394

[27] N. A. Dundon , A. H. Al Ghazwi , M. G. Davey , and W. P. Joyce , “Rectal Cancer Surgery: Does Low Volume Imply Worse Outcome‐a Single Surgeon Experience,” Irish Journal of Medical Science 192, no. 6 (2023): 2673–2679, 10.1007/s11845-023-03372-z. 37154997 PMC10165279

[28] K. B. Stitzenberg , E. R. Sigurdson , B. L. Egleston , R. B. Starkey , and N. J. Meropol , “Centralization of Cancer Surgery: Implications for Patient Access to Optimal Care,” Journal of Clinical Oncology 27, no. 28 (2009): 4671–4678, 10.1200/jco.2008.20.1715. 19720926 PMC3039919

[29] M. Huguet , “Centralization of Care in High Volume Hospitals and Inequalities in Access to Care,” Social Science & Medicine 260 (2020): 113177, 10.1016/j.socscimed.2020.113177. 32712556

[30] A. A. Morrow , A. McCombie , F. Jeffery , C. Frampton , and T. Hore , “Centralisation of Specialist Cancer Surgery: An Assessment of Patient Preferences for Location of Care in the Upper South Island of New Zealand,” ANZ Journal of Surgery 93, no. 9 (2023): 2180–2185, 10.1111/ans.18643. 37525374

