# Supplementary material for: How Is Colorectal Cancer Care Impacted by Global Crisis in Contrasting Healthcare Systems?—A Descriptive Study From Scotland and Switzerland During the COVID‐19 Pandemic
Source: World J Surg. 2026 Mar 2;50(4):818–28. doi: 10.1002/wjs.70294 (PMC13070440; doi:10.1002/wjs.70294)
Supplement: Supplementary file 1 — Supporting Information S1 [file WJS-50-818-s001.docx]

**How is colorectal cancer care impacted by global crisis in contrasting healthcare systems ?**

**- A descriptive study from Scotland and Switzerland during the COVID-19 pandemic**

B. Wiesler^1^, M. Worni^1,2^, P. Studer^2^, J.-M. Gass^3,4^, J. Metzger^3^, M. Hartel^5^, C. Nebiker^5^, R. Rosenberg^6^, R. Galli^6^, L. Eisner^7^, C. Andreou^7^, U. Zingg^8^, D. Stimpfle^8^, C. T. Viehl^9^, A. Müller^9^, B. Müller^1^, K. Denhaerynck^10^, P. Hall^11^, C. Gallagher^11^, P. Karunaratne^11^, C. Lilley^11^, M. Zuber^1,12^, H. Paterson^13*^, M. von Strauss und Torney^1,12*^

^1^Clarunis, Department of Visceral Surgery, University Digestive Health Care Center, St. Clara Hospital and University Hospital Basel, Switzerland

^2^Stiftung Lindenhof I Campus SLB, Swiss Institute for Translational and Entrepreneurial Medicine, Berne, Switzerland

^3^Department of Visceral Surgery, Cantonal Hospital of Lucerne, Lucerne, Switzerland

^4^Department of Health Sciences and Medicine, University of Lucerne, Lucerne, Switzerland

^5^Department of Visceral Surgery, Cantonal Hospital of Aarau, Aarau, Switzerland

^6^Department of Visceral Surgery, Cantonal Hospital of Basel-Land, Liestal, Switzerland

^7^Department of Surgery, Cantonal Hospital of Olten, Olten, Switzerland

^8^Department of Visceral Surgery, Spital Limmattal, Schlieren, Switzerland

^9^Department of Surgery, Spitalzentrum Biel, Biel, Switzerland

^10^Department of Public Health, University of Basel, Basel, Switzerland

^11^Edinburgh Cancer Research Centre, University of Edinburgh, Edinburgh, Scotland

^12^St. Clara Research Ltd., St. Clara Hospital, Basel, Switzerland

^13^Department of Colorectal Surgery, Western General Hospital, Edinburgh, Scotland

^*^The authors have contributed equally to the current work and share last authorship.

**Corresponding author**

PD Dr. med. Marco von Strauss und Torney

Clarunis, Department of Visceral Surgery, University Digestive Health Care Center, St. Clara Hospital and University Hospital Basel

Spitalstrasse 21, CH 4031 Basel

St. Clara Research Ltd., St. Clara Hospital, Basel

Email: marco.vonstrauss@clarunis.ch

ORCID ID https://orcid.org/0000-0003-1872-2397

| **Supplementary Figures and Tables** |  |
| --- | --- |
| ESM_Figure 1 | *pag. 3* |
| ESM_Figure 2 | *pag. 4* |
| ESM_Figure 3 | *pag. 5* |
| ESM_Table 1 | *pag. 6* |
| ESM_Table 2 | *pag. 7* |
| ESM_Table 3  ESM_Table 4  ESM_Table 5 | *pag. 8*  *pag. 9*  *pag.10* |

**ESM_Figure 1: Flowchart illustrating the process of data linkage (SCAN)**

**
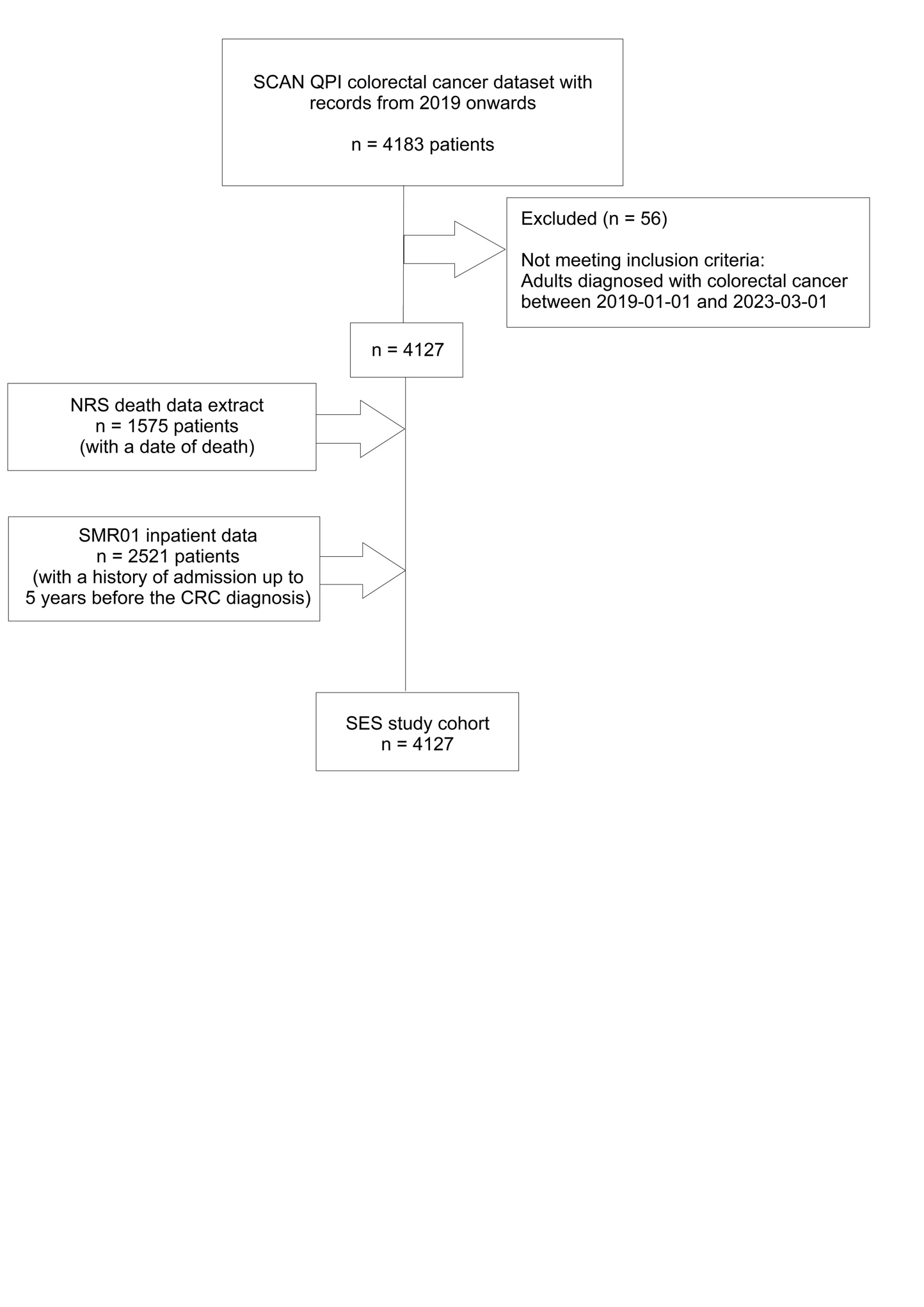
**

SCAN: South-East Scotland Cancer Network; QPI: Quality Performance Indicators; NRS: National Records of Scotland; SMR: Scottish Morbidity Records; CRC: Colorectal cancer; SES: South-East Scotland.

**ESM_Figure 2: Time from diagnosis to first treatment in (A) elective patients and (B) emergency patients in Scotland and Switzerland**


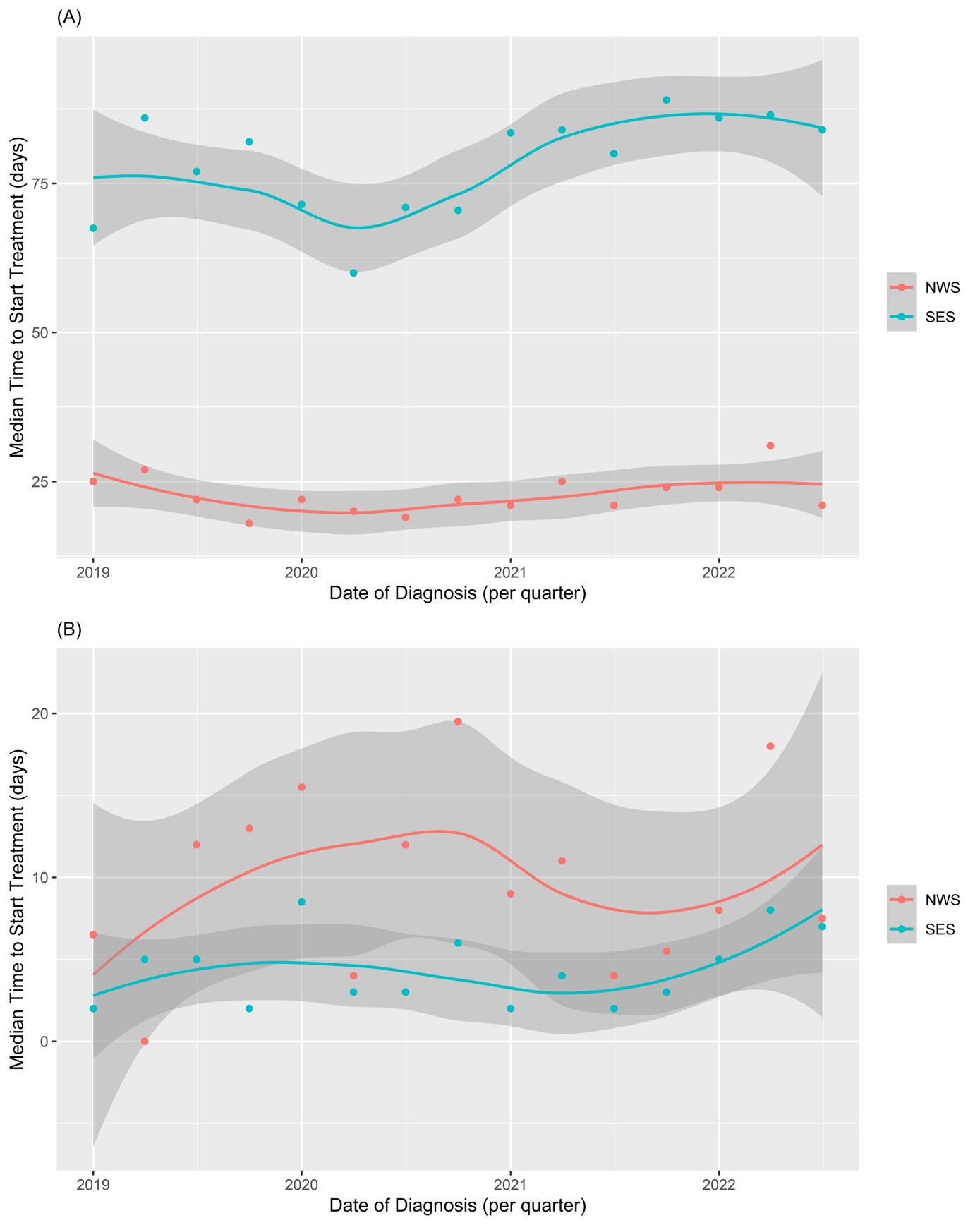


**ESM_Figure 3: (A) T-Stage at time of diagnosis (B) N-Stage at time of diagnosis and (C) M-Stage at time of diagnosis displayed for patients who were diagnosed for colorectal cancer between January 2019 and December 2022 in Scotland and Switzerland.**

(A) (B) (C)


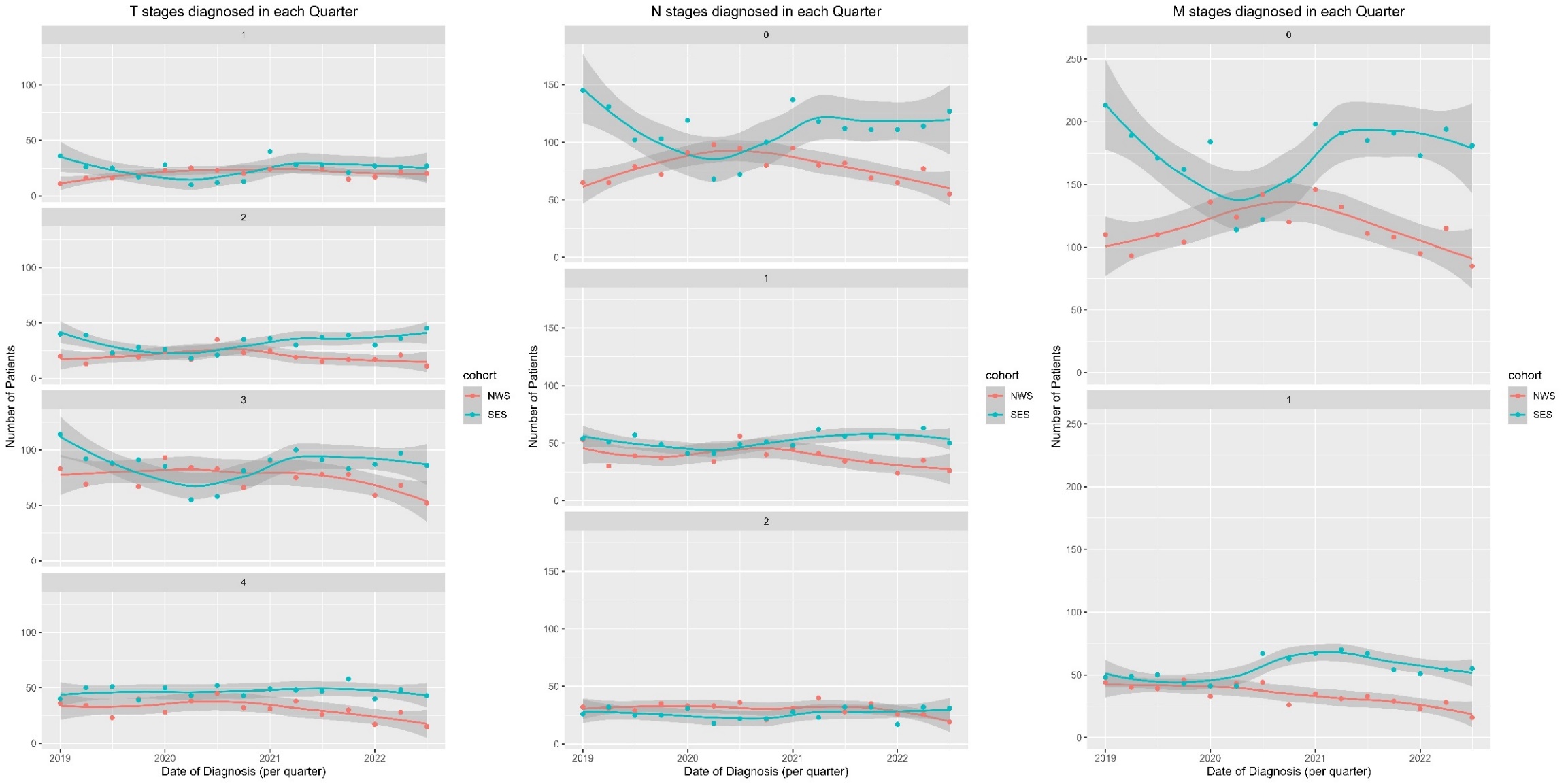


**ESM_Table 1: Patient and oncological characteristics subdivided by period.**

|  | | | **Pre Lockdown** | | | | **During Lockdown** | | | **Post Lockdown** | |
| --- | --- | --- | --- | --- | --- | --- | --- | --- | --- | --- | --- |
|  | | | **SES** group  (n=1247) | | **NWS** group  (n=808) | | **SES** group  (n=582) | | **NWS** group  (n=197) | **SES** group  (n=2298) | **NWS** group  (n=1613) |
| **Age** (Median, IQR) | | | 71 (63 - 80) | | 72 (61 - 80) | | 73 (64 - 81) | | 71 (59 - 79) | 72 (63 - 80) | 71 (61 - 79) |
| **Men** (n, %) | | | 682 (54.7%) | | 472 (58.6%) | | 302 (51.9%) | | 107 (54.3%) | 1214 (52.8%) | 930 (57.7%) |
| **Charlson Comorbidity Index** (n, %) | |  | |  | |  | |  | |  | |
| 0 | | | 419 (55.1%) | | 353 (49.6%) | | 186 (48.4%) | | 95 (53%) | 751 (56.5%) | 841(57.6%) |
| 1-2 | | | 183 (24.1%) | | 98 (14%) | | 96 (25%) | | 37 (21%) | 333 (25%) | 190 (13%) |
| ≥ 3 | | | 158 (20.8%) | | 260 (36.6%) | | 102 (26.6%) | | 48 (27%) | 246 (18.5%) | 430 (29.4%) |
| **Localisation** (n, %) |  |  | |  | |  | |  | |  | |
| Colon | | | 900 (72.2%) | | 526 (69.2%) | | 430 (73.9%) | | 129 (68.6%) | 1716 (74.7%) | 999 (65.5%) |
| Rectum | | | 347 (27.8%) | | 234 (30.8%) | | 152 (26.1%) | | 59 (31%) | 582 (25.3%) | 526 (34.5%) |
| **Neoadjuvant Therapy** (n, %) | | | 109 (8.7%) | | 126 (15.6%) | | 39 (7%) | | 27 (14%) | 183 (8%) | 251 (15.6%) |
| **Adjuvant Therapy** (n, %) | | | 245 (19.6%) | | 125 (15.5%) | | 105 (18%) | | 29 (15%) | 417 (18.1%) | 181 (11.2%) |
| **Palliative Therapy**  (n, %) | | | 149 (11.9%) | | 109 (13.5%) | | 96 (17%) | | 22 (11%) | 335 (14.6%) | 161 (10%) |
| **Type of the first Therapy** (n, %) |  |  | |  | |  | |  | |  | |
| Surgery | | | 764 (61.3%) | | 548 (67.8%) | | 350 (60.1%) | | 132 (67%) | 1386 (60.3%) | 1020 (63.2%) |
| Radiotherapy | | | 74 (6%) | | 25 (3%) | | 39 (7%) | | 10 (5%) | 129 (5.6%) | 45 (3%) |
| Chemotherapy | | | 0 (0%) | | 54 (7%) | | 0 (0%) | | 18 (9%) | 78 (3%) | 153 (9.5%) |
| Radiochemotherapy | | | 41 (3%) | | 81 (10%) | | 7 (1%) | | 13 (7%) | 49 (2%) | 181 (11.2%) |
| None | | | 368 (29.5%) | | 100 (12.4%) | | 186 (32%) | | 24 (12%) | 656 (28.5%) | 214 (13.3%) |
| **Any type of Therapy received** (n, %) | |  | |  | |  | |  | |  | |
| Surgery | | | 962 (77.1%) | | 657 (81.3%) | | 426 (73.2%) | | 158 (80.2%) | 1737 (75.6%) | 1290 (80%) |
| Radiotherapy | | | 90 (7%) | | 121 (15%) | | 47 (8%) | | 29 (15%) | 154 (6.7%) | 239 (14.8%) |
| Chemotherapy | | | 327 (26.2%) | | 156 (19.3%) | | 150 (25.8%) | | 35 (18%) | 595 (25.9%) | 357 (22.1%) |
| Radiochemotherapy | | | 49 (4%) | | 88 (11%) | | 8 (1%) | | 15 (8%) | 59 (3%) | 190 (11.8%) |
| None | | | 215 (17.2%) | | 100 (12.4%) | | 123 (21.1%) | | 24 (12%) | 424 (18.5%) | 201 (12.5%) |

**ESM_Table 2: Primary objective: Time from diagnosis to treatment and diagnosed Stages in the different periods of the pandemic.**

|  | **Pre-Lockdown** | | **During Lockdown** | | **Post-Lockdown** | |
| --- | --- | --- | --- | --- | --- | --- |
|  | **SES** group  (n=1247) | **NWS** group  (n=808) | **SES** group  (n=582) | **NWS** group  (n=197) | **SES** group  (n=2298) | **NWS** group  (n=1613) |
| **Time from diagnosis to first treatment (days)**  (Median, IQR) | 65 (35 -104) | 19 (6 - 36) | 59 (26 - 88) | 18 (7 - 35) | 76 (51 -104) | 21(8 - 37) |
| **Time from diagnosis to first treatment in elective patients (days)**  (Median, IQR) | 76 (48 -114) | 22 (10 - 39) | 68 (53 - 95) | 21 (11.5 - 37) | 83 (63 -109) | 23 (12 - 37) |
| **Time from diagnosis to first treatment in emergency patients (days)**  (Median, IQR) | 4 (1 – 24.5) | 8 (0 - 23) | 3.5 (1 - 18) | 9 (0 - 26) | 4 (1 - 33) | 10 (1 - 32) |
| **Time from diagnosis to surgical treatment (days)**  (Median, IQR) | 68 (32 -119) | 22 (7 - 64) | 59 (26 -93) | 19 (7.5 - 50) | 76 (48 -113) | 22 (8 - 52) |
| **UICC Stage** (n, %) |  |  |  |  |  |  |
| I | 242 (22.5%) | 139 (19.2%) | 83 (17%) | 32 (19%) | 453 (22.9%) | 319 (23.3%) |
| II | 319 (29.6%) | 186 (25.7%) | 126 (25.1%) | 55 (33%) | 505 (25.6%) | 339 (24.8%) |
| III | 289 (26.9%) | 200 (27.7%) | 129 (25.7%) | 37 (22%) | 512 (25.9%) | 429 (31.3%) |
| IV | 226 (21.0%) | 198 (27.4%) | 164 (32.7%) | 45 (27%) | 505 (25.6%) | 282 (20.6%) |
| **Number of patients diagnosed per UICC stage**  (Prevalence standardized per quarter) |  |  |  |  |  |  |
| I | 50 | 29 | 29 | 29 | 51 | 30 |
| II | 65 | 39 | 44 | 50 | 57 | 32 |
| III | 59 | 42 | 45 | 33 | 58 | 40 |
| IV | 46 | 41 | 57 | 41 | 57 | 26 |
| **T-Stage** (n, %) |  |  |  |  |  |  |
| 1 | 131 (13.4%) | 80 (11%) | 33 (8%) | 27 (16%) | 235 (13.4%) | 202 (14.8%) |
| 2 | 154 (15.8%) | 97(13%) | 70 (17%) | 15 (9%) | 311 (17.8%) | 194 (14.2%) |
| 3 | 465 (47.6%) | 389 (53.8%) | 181 (43.2%) | 91 (53%) | 791 (45.3%) | 681 (49.9%) |
| 4 | 226 (23.2%) | 157 (21.7%) | 135 (32.2%) | 40 (23%) | 411 (23.5%) | 287 (21.0%) |
| **N-Stage** (n, %) |  |  |  |  |  |  |
| 0 | 592 (60.5%) | 363 (50.8%) | 230 (54.6%) | 101 (58.0%) | 1011 (57.9%) | 739 (53.7%) |
| 1 | 251 (25.6%) | 198 (27.7%) | 131 (31.1%) | 34 (20%) | 491 (28.1%) | 351 (25.5%) |
| 2 | 136 (13.9%) | 153 (21.4%) | 60 (14%) | 39 (22%) | 245 (14%) | 285 (20.7%) |
| **M-Stage** (n, %) |  |  |  |  |  |  |
| 0 | 907 (80.1%) | 540 (73.2%) | 372 (69.4%) | 130 (74.3%) | 1612 (76.1%) | 1124 (79.9%) |
| 1 | 226 (19.9%) | 198 (26.8%) | 164 (30.6%) | 45 (26%) | 505 (23.9%) | 282 (20.1%) |

UICC: Union for International Cancer Control; SES: South-East Scotland; NWS: North-West Switzerland; IQR: Interquartile Range

**ESM_Table 3: Secondary objectives: The number of diagnosed and treated patients, the 30d mortality rates and the stoma rates in the different periods of the pandemic**.

|  | **Before Lockdown** | | **During Lockdown** | | **After Lockdown** | |
| --- | --- | --- | --- | --- | --- | --- |
|  | **SES** group  (n=1247) | **NWS** group  (n=808) | **SES** group  (n=582) | **NWS** group  (n=197) | **SES** group  (n=2298) | **NWS** group  (n=1613) |
| **Number of patients diagnosed per quarter**  (Median, IQR) | 256 (253 - 259) | 164 (164 - 167) | 203 (186 - 218) | 186 (186 - 186) | 270 (259 - 284) | 176 (159 - 193) |
| **Number of patients diagnosed**  (Prevalence standardized per quarter) | 255 | 168 | 203 | 178 | 259 | 151 |
| **Number of treated patients per quarter** (Median, IQR) | 177 (171 - 190) | 159 (142 - 167) | 152 (150 - 154) | 196 (194 - 199) | 188 (171 - 200) | 205 (195 - 211) |
| **Number of patients treated**  (Prevalence standardized per quarter) | 211 | 147 | 160 | 156 | 211 | 132 |
| **Number of patients starting the first**  **treatment per quarter**  (Median, IQR) | 173 (167 - 178) | 132 (122 - 138) | 135 (132 - 138) | 149 (146 - 152) | 177 (166 - 193) | 162 (150 - 166) |
| **30-day mortality rate**  (n, %) | 54 (4%) | 15 (2%) | 46 (8%) | 2 (1%) | 148 (6%) | 26 (2%) |
| **30-day mortality**  (Prevalence standardized per quarter) | 11 | 3 | 16 | 2 | 17 | 2 |
| **Stoma formation rate**  **All stoma** (n, %) | 255 (20.4%) | 220 (27.2%) | 129 (22.2%) | 47 (24%) | 479 (20.8%) | 425 (26.3%) |
| **Stoma rate in colonic cancer** (n, %) | 89 (10%) | 77 (15%) | 64 (15%) | 17 (13%) | 192 (11.2%) | 149 (14.9%) |
| **Stoma rate in rectal cancer** (n, %) | 166 (47.8%) | 142 (60.7%) | 65 (43%) | 30 (51%) | 287 (49.3%) | 269 (51.1%) |
| **Stoma formation rate**  **permanent stoma** (n,%) | 144 (11.5%) | 45 (6%) | 83 (14%) | 5 (3%) | 248 (10.8%) | 88 (6%) |
| **Permanent stoma in colonic cancer** (n,%) | 60 (7%) | 18 (3.4%) | 46 (10.7%) | 3 (2%) | 100 (5.8%) | 47 (5%) |
| **Permanent stoma in rectal cancer** (n,%) | 84 (24.2%) | 27 (12%) | 37 (24%) | 2 (3%) | 148 (25.4%) | 34 (7%) |

SES: South-East Scotland; NWS: North-West Switzerland; IQR: Interquartile Rang

**ESM_Table 4: Univariable and multivariable logistic regression analysis for being diagnosed with advanced tumor stage (UICC IV) in Scotland (SES group) and Switzerland (NWS group) – Complete case analysis.**

|  |  | | | | | | | |
| --- | --- | --- | --- | --- | --- | --- | --- | --- |
| **SES group** | | **n** | **Unadjusted**  **Odds ratio** | **95% CI** | **p-value** | **Adjusted**  **Odds ratio** | **95% CI** | **p-value** |
| **Age** |  | 4127 | 1.01 | 1.00 – 1.01 | **0.018** | 0.99 | 0.98 – 1.00 | **0.042** |
| **Sex** | Female  Male | 1929  2198 | Reference  0.99 | 0.85 – 1.15 | 0.89 | - | - | - |
| **CCI** | 0  1-2  ≥ 3 | 1356  612  506 | Reference  0.98  4.03 | 0.75 – 1.27  3.17 – 5.13 | 0.88  **<0.001** | Reference  0.73  1.76 | 0.46 – 1.13  1.18 – 2.59 | 0.17  **0.005** |
| **Localization** | Colon  Rectum | 3046  1081 | Reference  0.75 | 0.62 – 0.89 | **0.001** | Reference  1.40 | 0.91 – 2.11 | 0.12 |
| **Presentation** | Elective  Emergency | 2357  555 | Reference  4.95 | 3.90 – 6.29 | **<0.001** | Reference  4.95 | 3.49 – 7.07 | **<0.001** |
| **Pandemic** | Pre-lockdown  Lockdown  Post-lockdown | 1247  582  2298 | Reference  1.82  1.29 | 1.44 – 2.31  1.08 – 1.55 | **<0.001**  **0.004** | Reference  1.78  1.54 | 1.11 – 2.85  1.06 – 2.27 | **0.016**  **0.025** |
| **Time to first  treatment** | | 3930 | 0.99 | 0.99 – 0.99 | **<0.001** | - | - | - |
|  |  | | | | | | | |
| **NWS group** | | **n** | **Unadjusted**  **Odds ratio** | **95% CI** | **p-value** | **Adjusted**  **Odds ratio** | **95% CI** | **p-value** |
| **Age** |  | 2499 | 0.99 | 0.98 – 1.00 | **0.039** | 0.98 | 0.97 – 0.99 | **<0.001** |
| **Sex** | Female  Male | 1105  1509 | Reference  0.93 | 0.76 – 1.14 | 0.49 | - | - | - |
| **CCI** | 0  1-2  ≥ 3 | 1289  325  738 | Reference  1.00  5.44 | 0.66 – 1.48  4.27 – 6.97 | 0.10  **<0.001** | Reference  1.76  9.33 | 1.07 – 2.87  6.65 – 13.35 | **0.024**  **<0.001** |
| **Localization** | Colon  Rectum | 1654  819 | Reference  0.78 | 0.62 – 0.97 | **0.027** | Reference  0.88 | 0.65 – 1.18 | 0.39 |
| **Presentation** | Elective  Emergency | 1456  559 | Reference  2.28 | 1.81 – 2.88 | **<0.001** | Reference  2.39 | 1.80 – 3.18 | **<0.001** |
| **Pandemic** | Pre-lockdown  Lockdown  Post-lockdown | 808  197  1613 | Reference  0.92  0.70 | 0.62 – 1.34  0.57 – 0.87 | 0.67  **0.001** | Reference  0.88  0.64 | 0.52 – 1.46  0.48 – 0.85 | 0.62  **0.002** |
| **Time to first  treatment** | | 2295 | 1.00 | 1.00 – 1.01 | **<0.001** | - | - | - |

SES: South-East Scotland; NWS: North-West Switzerland; CI: Confidence Interval; CCI: Charlson Comorbidity Index

**ESM_Table 5: Univariable and multivariable logistic regression analysis for 30-day mortality after operation in Scotland (SES group) and Switzerland (NWS group) – Complete case analysis.**

|  |  | | | | | | | |
| --- | --- | --- | --- | --- | --- | --- | --- | --- |
| **SES group**  **(number of death=248)** | | **n** | **Unadjusted**  **Odds ratio** | **95% CI** | **p-value** | **Adjusted**  **Odds ratio** | **95% CI** | **p-value** |
| **Age** |  | 4127 | 1.05 | 1.04 – 1.07 | **<0.001** | 1.05 | 1.02 – 1.09 | **0.005** |
| **Sex** | Female  Male | 1929  2198 | Reference  0.86 | 0.66 – 1.11 | 0.23 | - | - | - |
| **CCI** | 0  1-2  ≥ 3 | 1356  612  506 | Reference  1.96  3.77 | 1.32 – 2.89  2.63 – 5.41 | **0.001**  **<0.001** | Reference  1.06  1.94 | 0.43 – 2.42  0.84 – 4.31 | 0.89  0.11 |
| **Localization** | Colon  Rectum | 3046  1081 | Reference  0.40 | 0.27 – 0.57 | **<0.001** | Reference  1.24 | 0.42 – 3.08 | 0.68 |
| **Presentation** | Elective  Emergency | 2357  555 | Reference  4.76 | 2.79 – 8.14 | **<0.001** | Reference  3.34 | 1.62 – 6.97 | **0.001** |
| **Neoadjuvant** | No  Yes | 3796  331 | Reference  0.13 | 0.03 – 0.35 | **0.001** | Reference  0.67 | 0.03 – 3.99 | 0.71 |
| **Pandemic** | Pre-lockdown  Lockdown  Post-lockdown | 1247  582  2298 | Reference  1.90  1.52 | 1.26 – 2.84  1.11 – 2.11 | **0.002**  **0.010** | Reference  1.25  2.24 | 0.39 – 3.90  1.01 – 5.70 | 0.69  0.06 |
| **Time to first  treatment** |  | 3930 | 0.98 | 0.98 – 0.99 | **<0.001** | - | - | - |
|  |  | | | | | | | |
| **NWS group**  **(number of death=43)** | | **n** | **Unadjusted**  **Odds ratio** | **95% CI** | **p-value** | **Adjusted**  **Odds ratio** | **95% CI** | **p-value** |
| **Age** |  | 2499 | 1.05 | 1.02 – 1.09 | **0.001** | 1.03 | 1.00 – 1.06 | **0.030** |
| **Sex** | Female  Male | 1105  1509 | Reference  1.53 | 0.82 – 2.99 | 0.20 | - | - | - |
| **CCI** | 0  1-2  ≥ 3 | 1289  325  738 | Reference  2.00  3.58 | 0.69 – 5.20  1.81 – 7.44 | 0.17  **<0.001** | Reference  1.47  2.71 | 0.47 – 4.51  1.21 – 6.90 | 0.50  **0.022** |
| **Localization** | Colon  Rectum | 1654  819 | Reference  0.26 | 0.09 – 0.61 | **0.005** | Reference  0.63 | 0.21 – 1.51 | 0.35 |
| **Presentation** | Elective  Emergency | 1456  559 | Reference  2.66 | 1.39 – 5.09 | **0.003** | Reference  1.59 | 0.81 – 3.11 | 0.17 |
| **Neoadjuvant** | No  Yes | 2214  404 | Reference  0.00 | 0 – 2.5×10^10^ | 0.99 | Reference  0.00 | 0 – 2.2×10^14^ | 0.99 |
| **Pandemic** | Pre-lockdown  Lockdown  Post-lockdown | 808  197  1613 | Reference  0.54  0.87 | 0.08 – 1.94  0.46 – 1.68 | 0.42  0.66 | Reference  0.72  1.04 | 0.11 – 2.70  0.52 – 2.16 | 0.67  0.90 |
| **Time to first  treatment** |  | 2295 | 1.00 | 1.00 – 1.01 | 0.06 | - | - | - |

SES: South-East Scotland; NWS: North-West Switzerland; CI: Confidence Interval; CCI: Charlson Comorbidity Index
